# First-Line Managers’ Experiences of Working with a Structured Support Model for Systematic Work Environment Management

**DOI:** 10.3390/ijerph17113884

**Published:** 2020-05-30

**Authors:** Fredrik Molin, Therese Hellman, Magnus Svartengren

**Affiliations:** 1 Department of Medical Sciences, Occupational and Environmental Medicine, 752 37 Uppsala, Sweden; therese.hellman@medsci.uu.se (T.H.); magnus.svartengren@medsci.uu.se (M.S.); 2 IPF, the Institute for Organizational and Leadership Development at Uppsala University, 753 20 Uppsala, Sweden

**Keywords:** work environment, managerial leadership, systematic work environment management

## Abstract

*Background:* This paper describes the experiences of first-line managers when working with a structured support model for systematic work environment management in their work groups. First-line managers play a key part in influencing the work environment. *Methods:* In this study, a sample of managers implementing a structured support model, the Stamina model, in Swedish municipalities were interviewed. A total of 31 (*n* = 31) interviews were conducted at two time points during a one-year period. The collected data were analysed using a qualitative thematic approach. *Results:* The results showed that managers experienced discomfort when giving the responsibility of working with work environmental issues to employees. However, managers also experienced and were impressed by how well it worked in allowing employees to take on work environmental issues. Managers found that they balanced between being quiescent and, at the same time, actively monitoring progress in the work groups. *Conclusions:* The results from this study implicate that managers need to be sensitive to the needs and capacity of their work groups. The oracle in Delphi stated know yourself. We conclude: Know your group!

## 1. Introduction

Developing and maintaining good health and productivity in businesses requires a structured process that supports the development of good working environments [1]. In Sweden, the governmental provision in the systematic work environment management addresses employer’s legal requirement to investigate, carry out, and follow-up on activities in such a way that ill health and accidents at work are prevented and a satisfactory work environment is achieved (AFS 2001:1) [2]. Previous research has found that such provisions are an important incentive influencing the actions taken [3,4,5]. First-line mangers in Swedish organisations often have the delegated responsibility to assess and take actions to ensure a good work environment, which makes them important actors in this work. In this study, first-line managers are defined as managers responsible for daily operations and hence with authority over “budgeting/…/staffing decisions and accountability for business performance” [6] of a work unit or a work group.

One barrier for implementing an initiative focusing on the systematic work environment management is the lack of time in organisations. Previous research has found that daily operations are more highly prioritised than these kinds of work environment initiatives, even though the involvement in such initiatives might save time and increase the organisation’s productivity. In these cases, the first-line manager has a vital role because his or her prioritisations are also the prioritisation of the employees [7].

First-line managers also play a key part in areas other than those mentioned above. First-line managers, in general, have the position and the possibility to influence the work environment through their support to employees [8,9]. It is well-known that support provided by the first-line manager positively influences employees’ job satisfaction, work engagement [10], staff well-being [11] and perceived work environment [12]. Lack of supportive leadership is shown to be associated with lower self-rated health among employees [13,14]. Other important features of the work environment for employees are the demand, support, and decision latitude dimensions of work. Social and emotional support from managers are important factors contributing to employee health and well-being [9]. Nyberg and colleagues [15] show a link between managerial leadership (i.e., leadership performed by formally appointed managers [16]) and heart disease, and their study highlights the need for a closer look at managerial practice and its influence on employee’s work environment.

In this study, a structured support model for systematic work environment management has been used in order to support the first-line managers and employees in their recurrent work with the systematic work environment management. The model emphasises employee participation, and the process is intended to be employee-driven [17]. Previous results have shown that first-line managers were active in the process of working according to the model to a varying extent and also gave various preconditions to the employees [18]. This might be due to the lack of clarity in how much the first-line managers were supposed to be involved in the practical work in order not to take over the employee-driven process. Similar results have been found in other participatory organisational initiatives in which the top manager viewed it as supportive not to intervene and the first-line manager did not fully understand his/her part. Lack of managerial support and involvement was found to be an inhibiting factor in the performance of the intervention [7]. It is evident that first-line managers play a key part in implementing work environment initiatives, but they often feel uncertain about how to engage, especially when it comes to engaging with, and supporting, their employees [19]. New approaches are needed to clarify the first-line managers’ part in work environment initiatives in order to facilitate employee participation as this has been seen as a key component in effective organisational interventions [19,20].

The aim of this study is to describe first-line managers’ experiences working with an organisational work environment support model in their organisation.

## 2. Materials and Methods

This qualitative descriptive study is part of a larger project, focusing on the use and implementation of a support model for systematic work environment management, the Stamina model, in various municipalities across Sweden [17]. Previous studies within the project have focused on experiences of upper management [21], employees [22] and persons that have the role as facilitators in the provision of the model [18]. The project was approved by the Regional Ethics Committee in Uppsala, Sweden (Project reference number 2017/093).

### 2.1. Study Context: The Stamina Model

The Stamina model intends to provide structure and recurrent feedback to first-line managers and their employees in a systematic work environment management. One essential key feature in the model is employee participation. The Stamina model has its theoretical roots in group development theory, specifically the Integrated Model of Group Development (IMGD) [23,24] and has a participatory approach aimed at enhancing the work environment in participating work groups.

The Stamina model is a support model that includes a recurrent structure with three sessions a year. In the sessions, employees on the ground level of the organization work together to examine features of their common work environment. The number of participants is contingent of the size of the work groups in the municipal organization. The basis for group subdivision is that the participants in the session should share a common work environment and a common operational task or goal. The reported group size during the first-year sessions was between 11 and 30 participants. The first session (workshop) consists of: (1) reflections on the shared basic values, aims and goals of the work group; (2) reflections on the work group’s current work situation; (3) reflections on how the work group wants their work situation to be and (4) reflections on what actions can be taken to create the desired work situation. In the last step, the work group prioritises one activity which they want to focus on and creates an action plan based on a manual. The second and third sessions (follow-ups) include a review of previous action plans and creation of new action plans for the work group. For further information about the model, see [17].

The workshops in the model are delivered by facilitators working in the participating organisations. The two follow-ups are led by the facilitator or the first-line manager in the organisation, with support from the facilitator. Facilitators are appointed by their own organization and are often representatives of the central HR-function in the municipal organization. The appointed facilitator attends a two-day course to learn how to lead the workshop and how to support the participants in creating the action plans. The first-line managers do not receive any specific training, other than videos with instructions on how to run the sessions.

### 2.2. Participants

This study is conducted in 18 Swedish municipalities. Each municipality decided upon the extent to which they wanted to participate, as they decided how many employees (100–1500) would be involved in the initiative [17]. For an overview of participating municipalities and their regional location in Sweden, see Table 1.

The study population in this present study consisted of first-line managers for work groups in various municipalities in Sweden. The participants were included by using a purposive criterion sampling strategy [25], in collaboration with the project managers in the municipalities. The inclusion criterion for the participants was having the position as a first-line manager to a work group that used the Stamina model and the project manager in each municipality received information that one first-line manager from their municipality was to be included in the study. The project manager in each of the 18 participating municipalities received written and verbal information about the aim and procedure of the study, which they communicated to eligible participants. All participants gave their written informed consent. Since two first-line mangers left their position between the first and the second round of interviews, a total of 20 participants (*n* = 20: 14 women and 6 men) were included in this study. For further information about the participants, see Table 2.

### 2.3. Data Collection

A total of 31 interviews (*n* = 31) were conducted for the purposes of this study. Interviews were conducted at two different time-points, approximately nine months apart. The first set of interviews took place after the first session in the Stamina model had been performed by the groups. The second round of interviews took place approximately nine months later, i.e., when three sessions using the Stamina model had been performed. Eleven participants were interviewed on both occasions. In two municipalities, the first-line manager changed work between the interviews, and the new manager was interviewed on the second data collection point. Another five first-line managers were only interviewed in the first round of interviews, since their organisation did not keep the planned schedule for the model and thus had not performed all sessions at the time of the second data collection. In the first round of interviews, 18 interviews were conducted, in the second round of interviews, 13 interviews were conducted. Thus, in total, 31 interviews were conducted for the purpose of this study, see Table 3.

The interviews were conducted on-site in the municipalities by one of the authors and a research assistant, both with extensive experience in conducting research interviews. All interviews lasted between 45–60 min, were digitally recorded and thereafter transcribed verbatim. The interviews were based on a semi-structured interview guide [26], focusing on how the first-line managers experienced the use of the Stamina model and how this work influenced their employees’ work situation. The first interview covered the actual performance of the session and how this was experienced. The second interview focused on how the work based on the model was maintained in the time between the sessions and how the work influenced the work situations. All themes were addressed using broad open-ended questions. The broad questions were followed up with probing questions to gather in-depth information regarding the participants’ experiences and reflections.

### 2.4. Data Analysis

The material was analysed using a thematic approach [27] and stored and organised using the qualitative software NVivo 12, 2018 (QSR International Pty Ltd., https://www.qsrinternational.com/nvivo-qualitative-data-analysis-software/support-services/faqs). The analysis process started by thoroughly reading all the transcripts several times to grasp the material. In the initial phase of the analysis, all transcribed interviews were coded line-by-line [28]. Thereafter, the codes were compared to each other (in each interview separately) to sort the initial codes into broader segments, resulting in tentative categories. Alongside the process of merging codes into tentative categories, memos were continuously written that explored the categories and documented the researchers’ reflections. In the third step, the tentative categories and memos from each interview were compared and compiled. The first two interviews were analysed by two researchers to increase credibility of the findings. During the whole analysis, the researchers went back and forth between the material produced and the interview transcripts to ensure that the findings were grounded in data and not extensively influenced by the researchers’ pre-understandings. Furthermore, the preliminary findings were discussed among all authors of the study and an expert panel. These experts were the coordinators in the municipalities, thus having extensive knowledge about the municipalities and the model. They recognised the findings and could relate it to their own context, which are aspects of this study in order to strengthen its credibility [29].

## 3. Results

The findings present the first-line managers’ perspective when working with the Stamina model. The thematic analysis identified four categories.

### 3.1. Knowing about the Model in Order to Inform Others

The first-line managers described that they were assigned a small and non-active part in the beginning of the work process based on the Stamina model. This resulted in some consequences for the implementation of the model. Firstly, the first-line managers described that they did not know much about the process themselves as they did not engage the group in the first session. This first session was led by the facilitators in the organisations, who had undergone a two-day training in delivering the session. This meant that the first-line managers had limited information about the work based on the model and thus found it difficult to explain the aim and work process of the model to their employees. Even though the first-line managers expressed that they had the will and ambition to prepare their employees for this work, they found that they did not have the possibility.

*“I had presented the Stamina model, but I didn’t know much about it myself. So, I could really just report what I had been told and then I invited [the facilitator] to a staff meeting to talk a little more about it.”*

Some first-line managers also expressed that they were quite new in their position and thus had limited experience of working with the systematic work environment management in general. This hampered their ability to understand the aim of the model and the thoughts behind how to work with the model. Other managers, who did not fully understand the purpose of the model, created their own understanding and used the model for other related issues, such as organisational development and to increase the operation’s productivity. Still, some first-line managers who consciously worked with systematic work environment management in their work groups and believed in the value of a good work environment in order to have well-functioning operations understood the focus of the Stamina model. These managers also described that they put a lot of emphasis and time on informing about the upcoming work with the model before starting the first session.

*“We raised it at a conference and in e-mail discussions; it was a project that the municipality has chosen, and that we were chosen to participate, and that I wanted to include my part of the organisation in this. So, it was more like they understood why they were being asked and selected and so on.”*

Furthermore, these managers were also aware of the efforts required after the first session to facilitate the long-term engagement that was needed and planned for using the model.

### 3.2. Being Quiescent Increases the Employees’ Initiatives

The first-line managers described that the Stamina model was a useful tool to facilitate the employees’ participation and engagement in work environmental aspects. They appreciated that the employees defined the issues, which needed to be handled and those who came with solutions in order to solve the identified problems. When the employees took this responsibility and the first-line managers were quiescent, they realised that the proposed solutions became confirmed and positively received by the employees. One first-line manager brought up an example in which she had proposed a solution before that had not been accepted; however, now when the employees were supposed to come up with a solution, the same idea was put forward again and was positively received. Most of the first-line managers were thus enthusiastic about this shift in being more passive and instead letting the employees identify and solve work environmental issues at the workplace.

*“It is also a big win that [the employees] are included now. I mean, it is they who write the activity plans, it is they who decide who should do what, and it is they who execute the plan. So that’s a win. There are many things that they feel the importance of changing and doing that I don’t need to be involved in. It’s good, it’s really good.”*

Even though they saw many positive effects of this shift, several of the first-line managers also described that they had to struggle to decrease the control and leave the ownership of the questions to the employees. This was an unfamiliar situation for them, and some felt a bit uncomfortable. It was somewhat difficult to balance their own responsibility and engagement in relation to their employees; nonetheless, they also expressed that the results of stepping back often ended up being very good. One manager said it was very good that the first session in the model was performed by another person as it became more difficult for the first-line manager to tell the employees what they should focus on.

Working with this model shifted responsibility from management level to employee level regarding work environment management. Having the mandate to identify problems and solutions was often a new experience for the employees. One manager stated that the employees seemed to appreciate the increased responsibility, but at the same time, were a bit surprised to be given it. Still, she experienced that they became more motivated to work with organisational development and work environment management, even those who were less enthusiastic in the beginning.

*“It was more challenging the first-time round to get acceptance of what it is that we are doing. A lot more questions about the study and about research and how to work.//Now, everyone has seen what we have achieved in the meantime. So, now it’s not about why we do this, but rather what will be the next step when we have coordinated, and the action plans have been implemented.”*

### 3.3. Being Active in Keeping the Work Going

In the same way as the first-line managers had difficulties knowing how much to be involved during the sessions, they also had various experiences of how much they needed to be and should be involved in the work of implementing the solutions into practice after the sessions. The managers described that during the first year of working with the model, the employees took on the task to analyse and identify problems well. The managers highlighted the importance of taking action to realise their plans in practice, which was new to many employees. Some managers experienced that the employees had difficulties to go from word to action and they experienced it as a difficult balancing act between, on the one hand, pushing the employees in their work with the action plans and on the other hand, letting them have their own process. One manager realised the importance of letting the employees be the owner of the process but, at the same time, experienced that there was a risk that their efforts would fade. This manager found a middle path and carefully followed the work with the action plans between sessions. She supported the employees through close management and provided assistance with resource allocation to make things happen. She thus only supported with resources, but at the same time, she was very clear with her employees that she expected the work to be completed within the time plan. Another manager did not feel that it was necessary to be active in this process with all employees as she felt that they were independent and mastered this work right from the start. However, this varied among the work groups within her responsibility.

*“Everyone is on board directly and there is nothing negative, everyone is solution focused, no one says: no, we don’t do that. //But in the other [group]… It is harder, of course, when you have a lot of new staff and quite young staff, then you are in the middle of everything and try to learn everything.”*

Most of the managers experienced time constraints and workload as barriers to the implementation of the action plans. They described that this work needed to be integrated as a natural process in the operations. However, it was difficult to find ways for this integration in the beginning of the process. One manager said that she deliberately had a dialogue with her employees about the potential gains from the efforts made in the work based on the model.

### 3.4. Focusing Early on the Small and Neutral Work Environmental Issues

Not being greatly involved in the identification of problematic situations was not just experienced positively by the first-line managers. Even though they recognised gains in terms of increased employee engagement, they also experienced frustration and disappointment to some extent regarding the kind of problems that came to light. The managers described that at the beginning of the process, the employees often chose issues that were minor and very easily solved, perhaps already at the sessions, instead of taking the opportunity to handle questions regarding more complex issues, such as relations, collaboration and communication. However, the managers also related that they respected the employees’ need to focus on the concrete issues as well.

*“That which came up in the action plans about what the employees did, they went into such small details. So, it felt like they wrote the action plan around it and then it was almost like they had planned and done it in a week. They didn’t write anything long-term in their action plans.”*

As the process went on, the first-line managers changed their opinions about the employees’ work in identifying and solving the problems. First, they themselves had started to see the value in dealing with the small and concrete issues as they realised that the small adjustments also generated big effects. Secondly, they described that the action plans increased in quality and that the employees took on more complex issues than in the beginning of the process. The first-line managers interpreted that this shift happened as the employees felt more secure in the way of working and started to learn from each other. However, they also experienced that in everyday life it was easy to fall back into old habits and land in a complaining instead of a solution-focused thinking. In this respect, they saw that they had an important role.

*“They have worked with very small things, I think, but they have achieved what they wanted. We also should take time to learn how to do it. We should work on what we can improve in the small things and this is what they have been actively working on, I think.”*

## 4. Discussion

The aim of this paper is to describe first-line managers’ experiences working with an organisational work environment support model. It is important to focus on managers’ actions in work environment research because leadership is found to promote subjective well-being and work engagement among employees in an organisation [8]. Several studies, both quantitively and qualitatively oriented, show a link between leader behaviour and actions and the subjective well-being of the employees [30,31]. Leadership behaviours that are defined in the realms of transformative leadership [32] are found to promote subjective well-being in organisations. Such behaviours focus on creating a climate of authenticity, personal consideration and motivation as well as participation among followers in an organisation or a work group [33]. The structured support model used in this study deliberately puts a lot of responsibility for action on the members of the work groups and thus forces the managers to take a step back. This means that managers are forced to become more passive than usual in order to give the members of the group a chance to actively participate and take responsibility for their actions and action plans. The findings in this study revealed that giving more responsibility to the employees was a new experience for many of the managers. Also, even though the first-line managers were positive to this shift, they brought up some challenges when shifting the initiatives and responsibilities towards their employees. They experienced that the employees liked the increased involvement in the work environment management but lacked concrete experience in order to take appropriate actions at the beginning of the process. It was then a balancing act for the first-line managers to support the employees on a relevant level. Interestingly, it should be noted that this study is conducted in Sweden. Thus, in the context of distributed leadership or Swedish management style [34], this approach could be expected to come more naturally for Swedish managers. However, the managers described a feeling of discomfort when not being direct in the work groups regarding the issue of work environment management even though working in the Swedish context. It is not clear whether managers in other managerial cultures than ours would be even more uncomfortable being forced into a more passive role than usual by not taking command of their work groups [35,36]. These results thus implicate that the structured support model used in this study might be highly dependent on the cultural context of the environment where the model is being implemented. 

Some managers found a balance between leaving the responsibility to the employees while still having the possibility to follow the structure and give support on a structural level instead of on the content and issues that were brought up. They used a strategy wherein they signalled to their group that the work environment management is important and that the groups need to continue to work with their action plans between sessions. Such signals have, in previous research, been found to be important to make the employees prioritise efforts of this character [7]. This was done either by giving frequent reminders to the groups or by creating opportunities for the members of the work groups to meet during work hours and discuss their action plans. This kind of supportive leadership actions [14,37] and non-commanding type of leader behaviour can also be considered to be a part of a Swedish management style [34].

Another factor that contributed to the difficulties of being quiescent was the managers’ frustration over the work groups developing action plans that were “safe” and small in the beginning. This, however, should be expected. The members of the work groups need to develop a sense of security and comfort with the procedures in the support model in their groups before they address more “hot” issues. The level of psychological safety [38] needs to be developed in the work groups before addressing sensitive issues. According to Wheelan [23], groups develop through distinct phases; therefore, it would be unexpected for work groups to address sensitive issues before establishing a sense of security and belonging in the group. The managers in this study seemed to be unaware of the fact that the work groups need to establish a sense of security and that they need to understand and learn the procedures of the support model. This is expected to take at least a year, before they can be fully productive in the new way of working with work environment issues. Members of the groups may also be unfamiliar with the procedures and the fact that they can influence and take command of the process in working with a systematic support model. In order to be a supportive manager in this respect, it is important to ensure that they have the necessary knowledge and realistic expectations of the initiative taken. If not, there is a risk that the engagement will decrease before results of the efforts are shown.

It is difficult to implement systematic work environment management, and managers often feel that the guiding provisions are either too abstract or too encompassing [39]. Another hampering factor for implementing systematic work environment management in the operations is that leadership actions in organisations are often characterised by “brevity, variety, and discontinuity” [40,41]. Hence, first-line managers will find it difficult to prioritise and make time for systematic work environment work. Managers in this study reported that time constraints and workload were two barriers for prioritising the work with the structured support model. These two barriers seem to be common in all the work groups. Organisational factors seemed to be important in this regard. Some of the managers had large work groups that were dispersed locally, whilst other managers had small work groups and a possibility to get to know all the members of the group. Wheelan [42] argues that group size and productivity are closely related and that larger groups (>9 members) perform significantly worse than smaller groups, in terms of productivity. Larger groups also tend to disintegrate into smaller (sub) groups. Several municipal organisations seem to be organised with far too many employees per manager. It is thus hardly possible for the managers to engage face-to-face with all the employees and less possible to support the work with a structured support model for work environment management. It is easy to blame the managers for not actively being involved in their work with systematic work environment management. However, we believe that the issue of organisational opportunities needs to be addressed. If the structure of the organisation is an “impossible” structure, then it is an issue for the upper-level management to solve.

### Strengths and Limitations

This study has a qualitative approach with two data collection points. This might be seen as a strength in research, with the aim to explore a work process that is ongoing during an extended period of time. The longitudinal design of this project made it possible to gather data from different points in time and to gain a deeper understanding of how the respondents experienced the use of the Stamina model for one year. Furthermore, it enabled the possibility to strengthen a relationship based on trust between the researcher and the informant, which adds to the credibility of the data. Nonetheless, it should be noted that due to high workload and staff turnover, it was not the same managers that were interviewed at the first and second occasion in all municipalities, which makes the longitudinal aspect less beneficial in some cases. On the other hand, staff turnover and changes are to be expected in real-life longitudinal organisation studies. A strength in this study is that most of the municipalities were represented by the same managers at both data collection points.

Interviews provide a snapshot of the real world that the participants are dealing with in everyday life. In this study, it would thus have been advantageous to follow the managers in their everyday practice by adopting a participatory research approach, e.g., mixed method, organisational ethnography or action research. Such approaches might be valuable to use in forthcoming studies investigating first-line mangers’ actions. Another limitation of the study is the possibility that the respondents of the study represent a positive bias towards the implemented support model, since they represent the municipalities that followed the intended time plan. Those that did not follow the intended time plan were therefore not included in the second round of interviews. However, the decision to drop out of the program, or delay its execution, did not solely rest on the first-line mangers, which means that the managers that stayed on were not necessarily positively inclined towards the support model.

## 5. Conclusions

The results from this study clearly show how important it is for a manager to know his or her work groups. When working with a structured support model, leadership actions need to be tailored to the needs and capacity of the work group. The study shows how advantageous it is when first-line managers manage to balance between being quiescent in their leadership and being more active in their leadership towards their work groups. Leaders need to give responsibility to others for actions and, at the same time, monitor the progress of the work groups. Being forced to take a step back as a leader of a group gives insight into employees’ ability to take on responsibility.

## Figures and Tables

**Table 1:** Information about Municipalities.

Information about Municipalities	Municipalities (*n* = 18) Mean (Range)
Number of citizens in the municipality	101,000 (9000–330,000)
Number of employees in the municipality	6500 (1000–25,000)
Location of the municipality	
-Northern Sweden	7
-Middle Sweden	3
-Southern Sweden	8
Classification of the municipality	
-Large cities and municipalities near large cities	9
-Medium-sized towns and municipalities near medium-sized towns	6
-Smaller town/urban areas and rural municipalities	3

**Table 2:** Information about Participants.

Information about Participants	Participants (*n* = 20) Mean (Range)
Gender (female/male)	14/6
Age	43 (29–60)
Years of working in the municipality	9 (1–25)
Years of working as a manager *	4 (1–11)
Number of employees **	36 (9–70)

* Data missing for two participants. ** Data missing from seven participants.

**Table 3:** Information about Performed Interviews.

	Number of Interviews	Number of Interviews
First and second round of interviews	11	11
Only first round	7	--
Only second round	--	2
Total number of interviews		31
